# A registry-based comparative analysis of antibiotic usage reporting for adult cattle on Danish dairy farms

**DOI:** 10.1186/s13028-024-00763-9

**Published:** 2024-08-29

**Authors:** Maj Beldring Henningsen, Jeanette Kristensen, Carsten Thure Kirkeby, Søren Saxmose Nielsen

**Affiliations:** Department of Veterinary and Animal Sciences, Faculty of Health and Medical Sciences, University of Copenhagen, Grønnegårdsvej 8, 1870 Frederiksberg C, Denmark

**Keywords:** Animal daily dose (ADD), Registry data, Treatment frequency, Used daily dose (UDD)

## Abstract

**Background:**

Antimicrobial resistance (AMR) is a significant global health concern, necessitating the monitoring of antimicrobial usage (AMU). However, there is a lack of consensus on the standardized collection and reporting of AMU data in the veterinary field. In Denmark, the Danish Cattle Database (DCDB) contains treatment information on animal level, which allows counting of number of treatments carried out, used daily doses (UDD). The Danish VetStat database (VetStat) contains information on veterinary medicinal prescriptions at farm level and uses fixed standard doses of each product to calculate number of daily treatments, animal daily doses (ADD). This study aimed to compare two different numerators, UDD and ADD, used to describe AMU on Danish cattle farms, and estimate their correlation.

**Results:**

Routinely collected registry data from conventional dairy farms in Denmark for 2019 were used, including a total of 2,197 conventional dairy farms. The data from VetStat and the DCDB were aggregated and analysed, and treatment frequencies (TF) were calculated for both UDD and ADD, adjusting for farm size. Spearman correlation analysis and Bland–Altman plots were employed to assess the relationship and agreement between TF for ADD and UDD, respectively.

The results showed a high correlation between TF for ADD and UDD for most prescription groups, i.e., groups used to categorise antibiotics based on target organs. An exception is found for the Udder prescription group, where a systematic underreporting of UDD compared to ADD was observed. This discrepancy may be due to combination treatments, and potential missing or grouped registrations in the DCDB.

**Conclusions:**

Our UDD and ADD comparison yields valuable insights on farm-level AMU. We observe strong correlations between UDD and ADD, except for udder treatments, where some farms report only 1/3 UDD compared to ADD, indicating potential underreporting. Further investigations are needed to understand the factors contributing to these patterns and to ensure the accuracy and completeness of recorded information. Standardizing AMU data collection and reporting remains crucial to tackle the global challenge of AMR effectively.

## Background

Antimicrobial resistance (AMR) constitutes a global health challenge. With an established, yet complex, association between antimicrobial usage (AMU) and AMR [1], monitoring AMU is crucial. In this study, we focused exclusively on antibiotic usage, referring to it by the common abbreviation for antimicrobial usage, AMU. Ensuring standardised AMU data collection and reporting in the veterinary area is important but also challenging, and a consensus has not yet been reached [2–4]. We often see AMU reported with a numerator and a denominator [5]. The numerator describes the amount of antibiotics used. The denominator in AMU corrects for the size of the animal population e.g., Species Population Correction Unit (species PCU) or counted number of animals [4].

Typically, AMU numerators are defined as either count-based (number of treatments) or dose-based (number of calculated standard doses) [3, 6]. Count-based numerators are often the counted number of treatments, or the number of treatment courses and they do not require information about the amount of antimicrobial product used [3, 6]. The European Medicines Agency uses the term Defined Daily Doses for animals (DDDvet), which is a dose-based measure. DDDvet is the amount of an antimicrobial product to treat 1 kg of animal and is defined for each antimicrobial product, animal species, and route of administration based on the Summary of Product Characteristics (SPC) for antimicrobial products provided by medical companies and AMU data from multiple European countries [4, 7, 8].

An important step in the improvement of AMU quantification is to conduct studies comparing the implementation of several different numerators on the same AMU data [9]. This has been done for pigs in a study, which compared treatment frequencies (TF) using different numerators on AMU data from German pig farms [10]. TF is an important measure of AMU, indicating the number of treatments of one animal for one diagnosis per thousand animals. The numerators studied, Defined Daily Dose (DDD) and Used Daily Dose (UDD) represent count and use-based measures respectively [10, 11]. The calculated TF varied greatly between the two numerators with a 16% to 77% higher TF for DDD compared to UDD. This was due to the differences in the numerator (fixed doses used in the DDD measure and actual amount per daily treatment used in the UDD measure). Another study has suggested the Used Daily Dose per Animal (UDDA), a count-based measure, as the most accurate numerator for describing antibiotics administered to animals at farm level [3]. The accuracy of UDDA depends on the specific objective, whether it aims to capture actual number of animals treated, the antibiotic consumption, or the amount of antibiotics released into society, considering factors such as waste management practices.

In Denmark, the national database on prescription medicine, the Danish VetStat database (VetStat), contains detailed information on all antibiotics sold for use in Danish cattle and other animal species [12]. Sale of prescription medicine is always authorised by a veterinarian [13]. In VetStat, AMU is reported with a dose-based numerator Animal Daily Doses (ADD). The denominator is the number of animals per day at farm-level reported by age groups based on the animals’ age and calving status. Each age category has an assigned standard weight for one animal, e.g. 600 kg for adult cattle [14]. ADD is assigned per product primarily based on SPCs and the methods are outlined in the Danish legislation [15]. In official reports from the Danish Veterinary and Food Administration (DVFA), AMU is often reported as number of ADDs per 100 animals per day within age groups i.e., percent animals treated per day (ADD100) [15]. AMU in VetStat is further specified by reporting used ADDs by prescription groups. The prescription group corresponds to the organ system for which a given product is prescribed for treatment e.g., airways or gastro-intestinal tract [13].

Danish cattle farmers and veterinarians have a legal obligation to register the use of prescription medicine for production animals with identification of the animal(s) treated, along with the date the treatment occurred, diagnosis and product used [13, 16]. This information leads to knowledge of number of individual animals treated for one diagnosis for 1 day i.e., Used Daily Doses (UDD). Treatment data are generally entered into the Danish Cattle Database (DCDB) administered by SEGES (Aarhus N, Denmark), an independent research and innovation company promoting sustainable agricultural and food production. On the rare occasion where reporting of treatment data to the DCDB does not occur, the registrations should be available in another format e.g., paper records.

Our objective was to compare nominators used to describe AMU in the two different databases (VetStat and DCDB) and estimate their correlation. We compared TF of ADD calculated based on registrations in the national surveillance data from VetStat and the TF of UDDs from the corresponding on-farm antibiotics records from the DCDB using the same denominator, which is number of animals per day. The results will be presented as ADD and UDD per 1000 animals per day, specifically referred to as TF_ADD_ and TF_UDD_.

## Methods

### Data collection

**Fig. 1:**
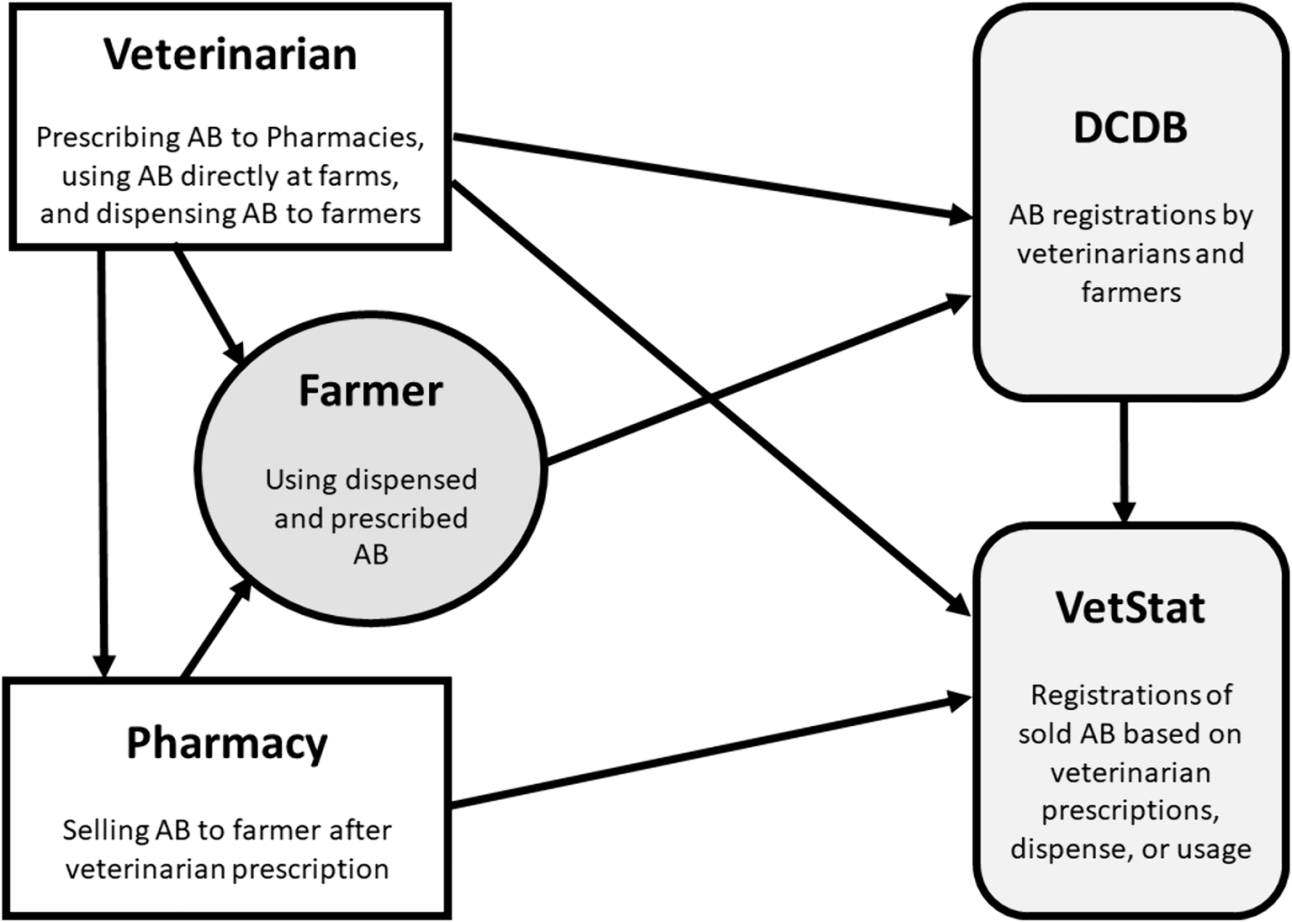
Data flows on recording of antibiotics (AB) in Danish cattle. The veterinarian and the farmer treat animals in Denmark, and either can register the on-farm AB use. Prescription medicine, all antibiotics included, is either used, dispensed, or prescribed by the veterinarian to the specific animal, or to the farm for a specific age group and diagnosis. Prescription medicine is sold by the pharmacy and sales data are reported by them to VetStat. Most Danish dairy farmers use software solutions linked to the Danish Cattle Database (DCDB). Data on medicine use, dispensing, or prescriptions by the veterinarian and medicine use by the farmer is entered into the DCDB. Data on the medicine used and dispensed by the veterinarian is reported to VetStat either directly or via the DCDB

We used routinely collected registry data from conventional dairy farms. Data on AMU were collected from the DCDB and VetStat for 2019 for each farm in the study population. Figure 1 represents the data flow to the two data registries.

### Nominators UDD and ADD

In the DCDB, the study unit considers one unique animal treated with antibiotics for one diagnosis for 1 day. The number of treatments can be summarised at farm-level to find the number of UDDs at farm-level

1 $$UDD = \mathop \sum \limits_{i = 1}^{n} T_{i}$$

where UDD is the farm-level number of daily treatments of one animal for one diagnosis for 1 day, and n is the number of observations, resulting inT_1-n_ being one animal treated for diagnosis on 1 day.

In VetStat, the study unit is the calculated standard dose for treatment of one adult animal with antibiotics for 1 day. Adult animals are defined as above 2 years of age or having calved and having a standardised body weight of 600 kg. For each product containing antibiotics, the specified amount required to treat one animal or 1 kg of animal for 1 day is detailed in VetStat. For each farm, the number of ADDs are calculated for each unique product sold to the farm separately. The ADDs are then summarised at farm-level

2 $$ADD = \mathop \sum \limits_{i = 1}^{n} ADD_{i} = \mathop \sum \limits_{i = 1}^{n} \frac{{AB_{n} }}{{D_{Species} *W_{Age } }}$$

where ADD is the number of animal daily doses sold to the farm, n the number of products, resulting in ADD_1-n_ being the farm-level number of animal daily doses sold to the farm of a product. D_Species_ is the fixed dose of the product to treat one kg of animal for the species given in doses, with g or mL corresponding to the units used for sold AB (AB_n_). W_Age_ is the standard weight of an animal in the age group within the species; i.e. 600 kg for adult cattle.

### Denominator “animal days”

The denominator used to correct for the population in our study is animal days. An "animal day" is defined as one animal present on one farm for 1 day. The number of animal days is counted on a monthly basis for each farm. Animal days are summed at farm-level for a year and divided by the number active days (i.e. days the farm has housed animals) to calculate the average number of animals present on the farm for the year when it is active and have animals at risk of treatment.

### Data manipulation

**Fig. 2:**
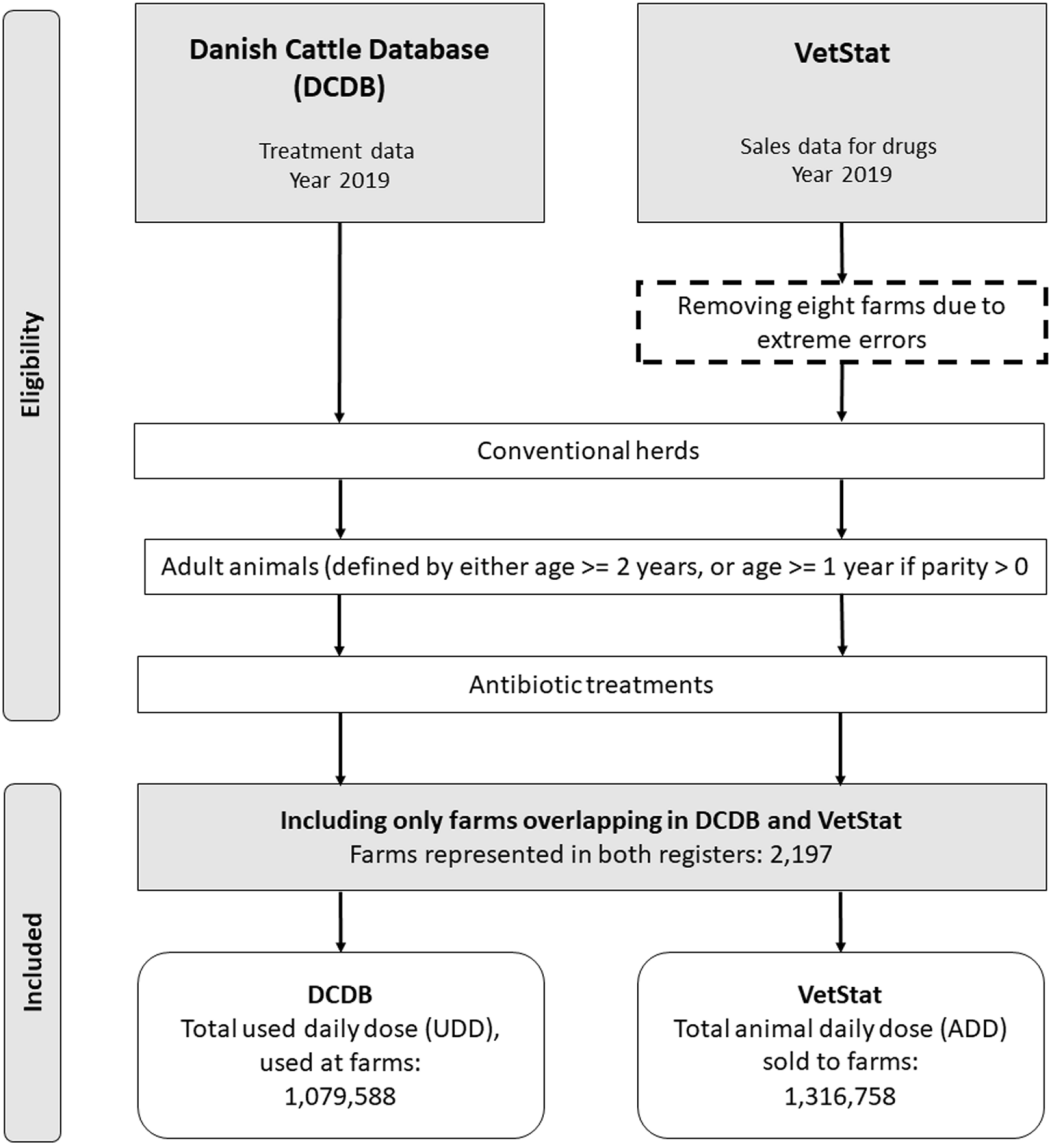
Representation of data selection from the Danish Cattle Database and VetStat. Treatment and sales data were included from both databases. Eight farms were removed due to extreme observations from the VetStat data. From the DCDB only records from conventional dairy farms holding VASC M2 (Veterinary Advisory Service Contract Module 2) were included. Records with products containing antibiotics were identified and included for each farm. Farms with records in both databases were included in the final dataset

We aggregated the number of UDDs and ADDs for 1 year, 2019. Figure 2 presents the data cleaning flow for DCDB and VetStat. The software R was used for all data handling [17]. Only conventional dairy farms holding a Veterinary Advisory Service Contract (VASC) with module 2 extension (M2) [18] and with treatment records in the DCDB were included in the study. From these farms only records of antibiotic treatments of cows and all cattle above 2 years of age were included. The corresponding data from VetStat were recorded sales of products containing antibiotics for cows and adult cattle for all Danish cattle farms. In the final study, 2,197 farms with data in both databases were included.

Eight farms were removed from the study data as we suspected a magnitude of error in the local report system for a specific product, e.g., 1 mL of a product was entered as one 100 mL bottle of the product.

All medicine reported to VetStat is assigned a prescription group. The prescription groups describe the specific organ systems for which the medicine is prescribed or used. These categories include “Reproduction and urogenital disorders”, “Udder disorders”, “Gastrointestinal disorders”, “Respiratory disorders”, “Joint, limb, hoof, central nervous system, and skin disorders”, and “Metabolism, digestion, and circulation disorders”. Additionally, a group “Other disorders” is used.

For each treatment in the DCDB, the diagnosis can be translated into the prescription groups used in VetStat. It is therefore possible to further group UDDs and ADDs by prescription group. All products are reported in both databases using Antibiotic groups in the Anatomical Therapeutic Chemical Classification System for veterinary medicinal products (ATCvet) codes. The ATCvet system is developed by the WHO Collaborating Centre for Drug Statistics Methodology at the Norwegian Institute of Public Health to provide a standardized classification system [19].

### Treatment frequency

We calculated treatment frequencies for UDDs and ADDs, respectively. Based on the UDDs, ADDs and information on “animal days” treatment frequencies per thousand were calculated using Eqs. 3 and 4 were used to correct for the effect of farm size in our analysis:

3 $$TF_{UDD} = \frac{UDD}{{n*d}}*1.000$$

4 $$TF_{ADD} = \frac{ADD}{{n*d}}*1.000$$

For Eq. 3, TF_UDD_ is treatment frequency given as number of daily treatments of one animal for one diagnosis per thousand animals, and UDD is the farm-level number of daily treatments of one animal for one diagnosis for one day. For Eq. 4 TF_ADD_ is the treatment frequency given as number of animal daily doses sold to the farm per thousand animals and ADD is the farm-level number of animal daily doses sold to the farm. For both Eqs. 3 and 4, n is the number of animals on average on the farm in 2019, and d is the number of days in 2019, where the farm had animals present.

### Statistical analysis

The results from the two databases were compared using a Bland–Altman plot [20]. When comparing two different methods of estimation, such as TF for ADD (TF_ADD_) and TF for UDD (TF_UDD_), Bland–Altman plots can be useful. It shows the difference between the two measurements against their mean, and for equivalent measures, we would expect to see scatter points at zero. We estimated a 95% confidence interval (CI) of the mean difference.

We utilized Spearman correlation analysis to assess the relationship between TF_ADD_ and TF_UDD_. Specifically, we examined the correlation grouped by farm and prescription group to gain insights into potential influencing factors. By applying Spearman correlation, which accounts for monotonic relationships, we aimed to capture non-linear associations and potential ordinal patterns in the data. This approach allowed us to explore how the correlation between the variables varies across different farm and prescription groups.

## Results

A total of 2,197 conventional Danish dairy farms were included in the final dataset after exclusion of ineligibles observations and farms only appearing in one of the registries. This was out of 3,057 unique CHR numbers in DCDB in 2019, including organic farms and farms that changed status (conventional to/from organic). Also excluded were the registrations at eight farms, as the prescribed antibiotics amounted to tons, suggesting a registration error.

Prescriptions were categorized in six different groups, providing a summary of the findings in Table 1. The Udder prescription group had the highest ADD (975,254) and TF_ADD_ (10,658) in VetStat and the highest UDD (776,438) and TF_UDD_ (8,077) in DCDB. For descriptive purposes, the top three diseases within each prescription group were identified in DCDB for 2019, except for the “Other” group. For udder-related prescriptions, mastitis was the most common (521,775 cases), followed by mastitis-related treatments at dry-off (156,053 cases), and acute mastitis (100,368 cases). In the reproduction and urogenital system group, Metritis (97,620 cases) was most prevalent, with Retained placenta (34,668 cases) and Vaginitis (3,517 cases) following. Hoof abscess (88,642 cases), Thick hock (30,070 cases), and Arthritis (7,268 cases) were the top diseases for joints, limbs, hooves, central nervous system, and skin. Pneumonia (13,124 cases) dominated the respiratory disorders, with other infections and lung worm being less frequent with less than 15 treatments each. The gastrointestinal group included primarily foreign body occurrence (3,767 cases), diarrhoea (1,717 cases), and displaced abomasum (944 cases).

**Table 1:** Prescriptions groups in VetStat and the Danish cattle database

Prescription group	Sum ADD	Sum TF_ADD_	Sum UDD	Sum TF_UDD_
Udder	975,254	10,658	776,438	8,077
Reproduction, urogenital system	168,311	1,591	135,752	1,254
Joints, limbs, hooves, central nervous system, skin	148,592	1,684	146,695	1,483
Respiratory disorders	14,091	164	13,059	123
Gastrointestinal	9,572	125	7,616	74
Other	938	14	28	0

Amount of animal daily doses (ADD) prescribed in the VetStat registry, and amount of unique used daily doses (UDD) of antibiotic in the Danish Cattle Database (DCDB) for adult Danish cattle in 2019. TF_ADD_ is the treatment frequency of animal daily doses sold to the farm per thousand animals and TF_UDD_ is the treatment frequency of daily treatments per thousand animals

**Fig. 3:**
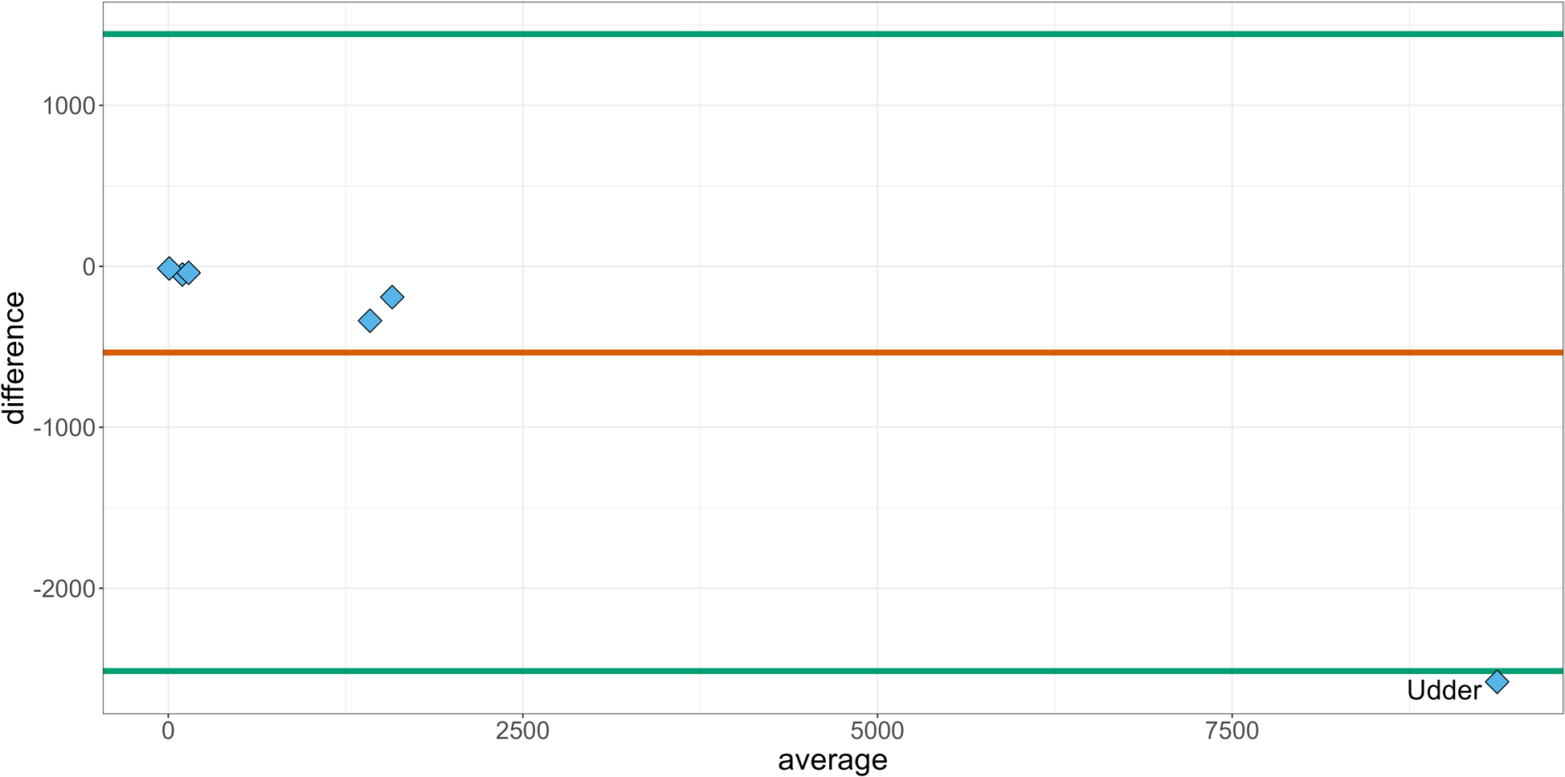
Bland–Altman plot of the differences in six prescription groups in treatment frequency of used daily dose (TF_UDD_) from the Danish Cattle Database and treatment frequency of animal daily doses (TF_ADD_) in the VetStat Database. The labelled outlier identifies the prescription group “Udder”, which include mastitis. In the two clustered plots the five remaining prescriptions groups are found. The two plots in the cluster to the right represents the prescription groups “reproduction, urogenital system” and “joints, limbs, hooves, central nervous, skin”. The three plots in the cluster to the left represents “respiratory disorders”, “gastrointestinal”, and “other”

The Bland–Altman plot in Fig. 3 comparing TF_UDD_ and TF_ADD_ in relation to six different prescription groups revealed that among the six groups, four of them showed a difference = 0, indicating a close agreement between TF_UDD_ and TF_ADD_ within those groups. The remaining two groups exhibited differences below zero, suggesting the mean of TF_UDD_ values to be lower than TF_ADD_ values across those groups. Furthermore, when considering the 95% CI, five of the groups fell within the CI limits, indicating acceptable agreement between TF_UDD_ and TF_ADD_. However, the Udder prescription group stood out as an exception, as it fell below the lower CI limit and exhibited the highest difference value.

**Fig. 4:**
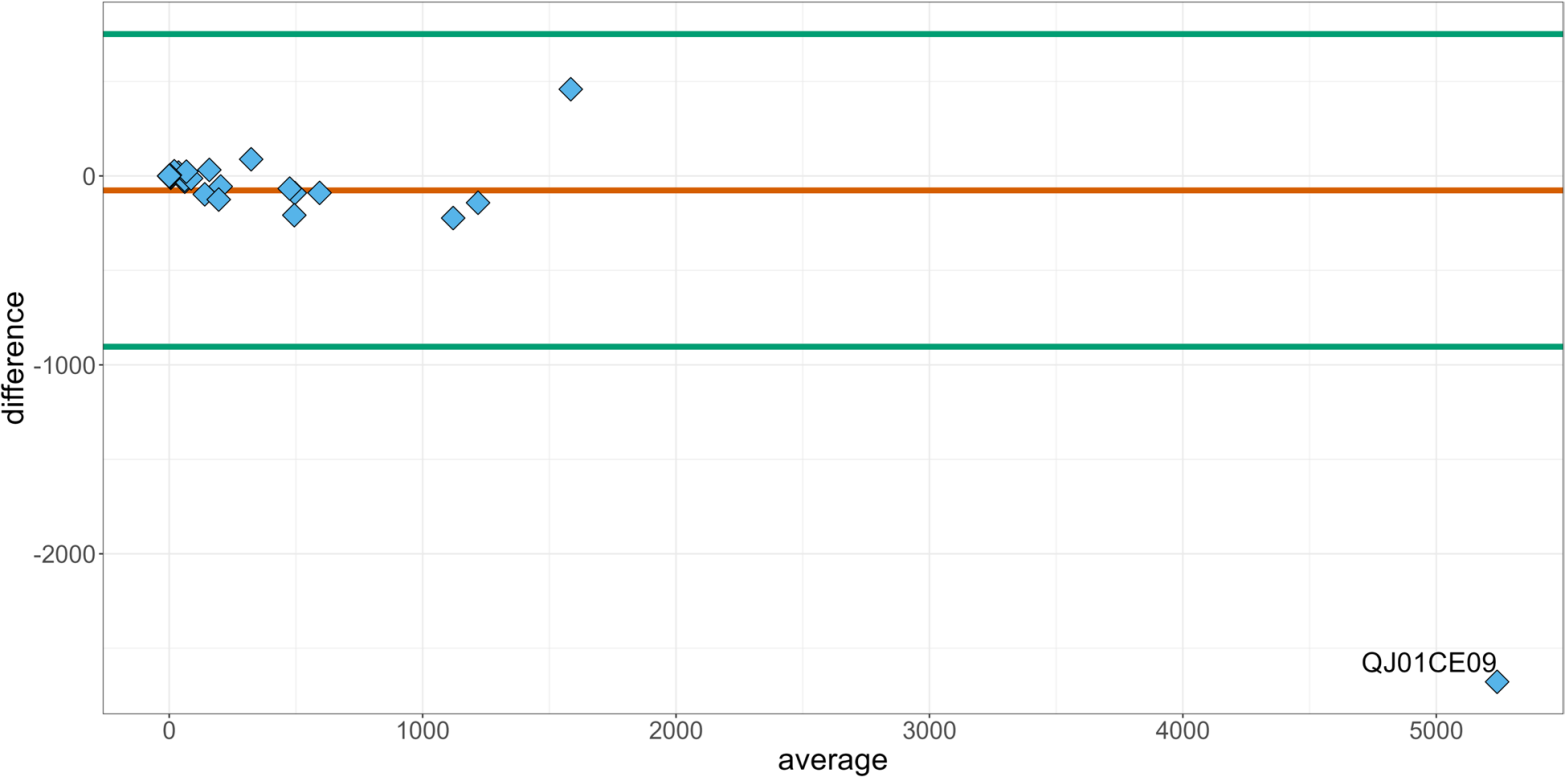
Bland–Altman plot of the differences per Antibiotic ATCvet group (Antibiotic groups in the Anatomical Therapeutic Chemical Classification System for veterinary medicinal product) in treatment frequency of used daily dose (TF_UDD_) from the Danish Cattle Database and treatment frequency of animal daily doses (TF_ADD_) in the VetStat Database. The labelled outlier identifies the ATCvet group QJ01CE09, which codes for the antibiotic procaine benzylpenicillin often used in dairy cattle for systemic treatment of gram-positive infections

The product with the ATCvet code QJ01CE09 (procaine benzylpenicillin) was also an outlier in the Bland–Altman plot for ATCvet groups (Fig. 4), with a high difference-value and a low average-value. This product can in cattle be used for treating gram-positive infections. Some observations were above the difference = 0 line, again indicating TF_UDD_ values exceeded TF_ADD_ values.

Table 2 presents a summary of TF per farm in 2019, as measured by the ADD in the VetStat database and UDD in DCDB. The VetStat database reported a total TF_AMU_ of 14,226 TF_ADD_, with a mean of 6.48 ADD per farm. The range of TF_AMU_ varied from a minimum of 0.03 TF_ADD_ to a maximum of 54.61 TF_ADD_. In the DCDB, the total TF_AMU_ was 11,012 TF_UDD_, with a mean of 5.01 TF_UDD_ per farm. The minimum AMU was 0.02 TF_UDD_, while the maximum reached 63.12 TF_UDD_.

**Table 2:** Summary of treatment frequency of antimicrobial usage (TF_AMU_) per farm in 2019

Database	Total TF_AMU_	Mean	Min	Q1	median	Q3	Max
VetStat^a^	14,226	6.48	0.03	3.18	5.87	9.03	54.61
DCDB^b^	11,012	5.01	0.02	1.68	4.33	7.45	63.12

^a^Results given as number of animal daily doses sold to the farm per thousand animals

^b^Results given as number of daily treatments of one animal for one diagnosis per thousand animals

Summary of treatment frequency of antimicrobial usage (TF_AMU_) per farm in 2019 in the VetStat Database (ADD) and the Danish Cattle Database (DCDB) (UDD)

**Fig. 5:**
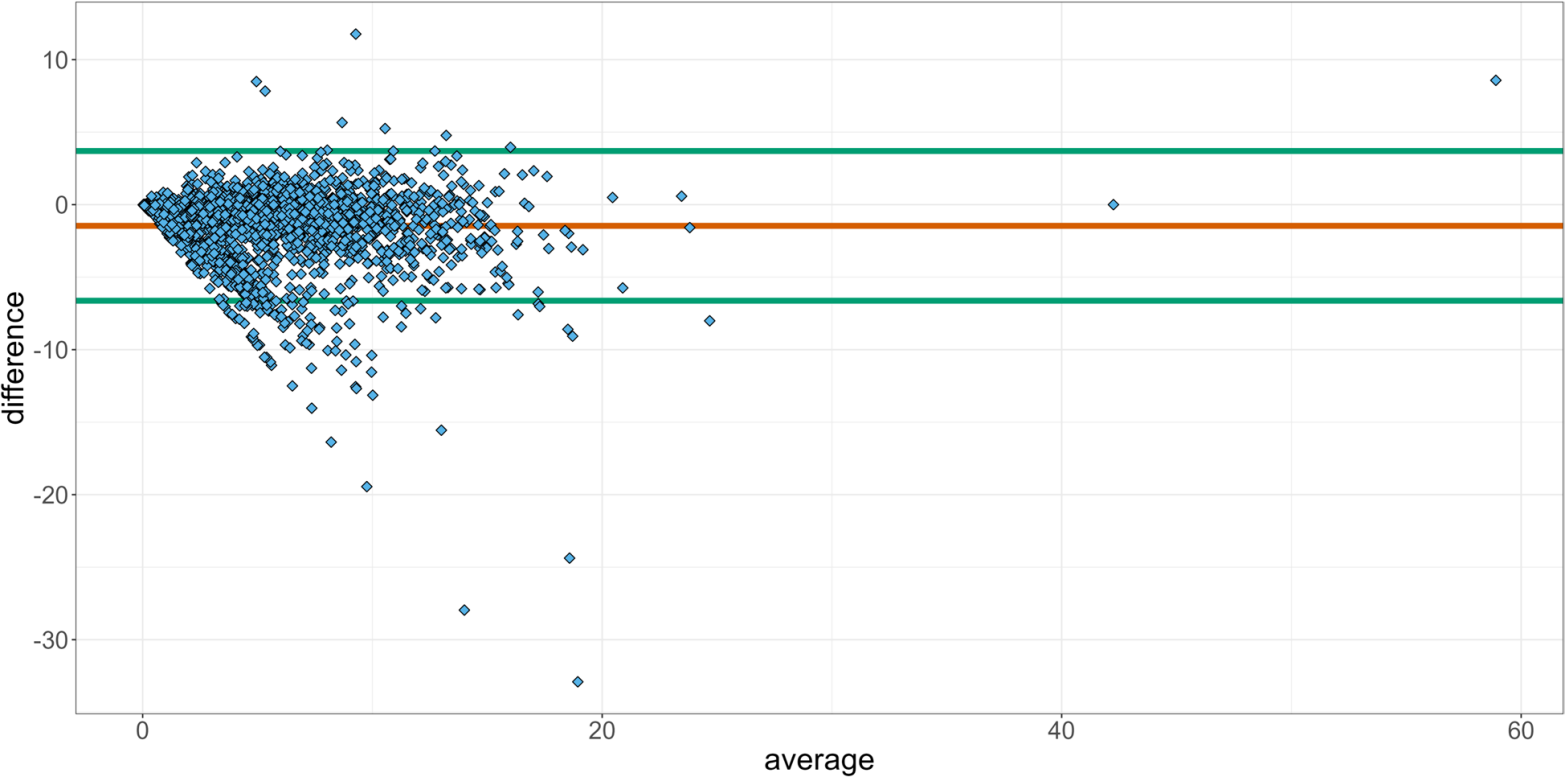
Bland–Altman plot of the differences for 2,197 Danish dairy farms in treatment frequency of used daily dose (TF_UDD_) and treatment frequency of animal daily doses (TF_ADD_) from respectively the Danish cattle database (DCDB) and the VetStat Database. The observations falling within the 95% CI limits provide insight into the precision of the mean difference estimation. These limits represent the range within which the true mean difference is expected to fall with 95% confidence. Regarding observations above the difference = 0 line, they indicate instances where the TF_UDD_ values exceed the TF_ADD_ values

In the Bland–Altman plot on farm level in Fig. 5, we observed that the majority of the 2,197 comparisons between TF_UDD_ and the TF_ADD_ were located below the difference = 0 line. This suggests a consistent tendency for the TF_UDD_ values to be lower than the corresponding TF_ADD_ values.

**Fig. 6:**
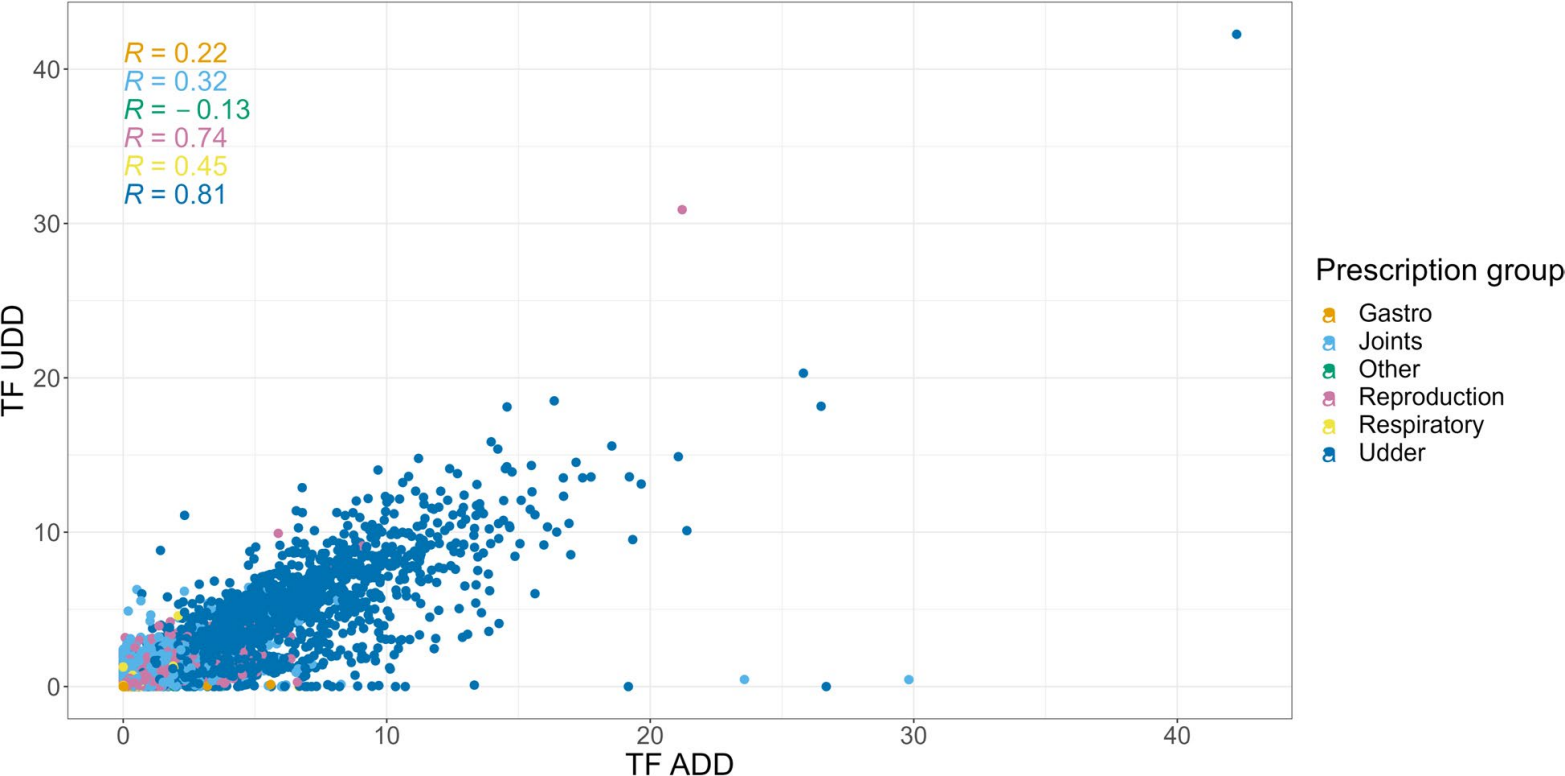
Spearman correlation plot of the farm and prescription group level differences in treatment frequency of used daily dose (TF_UDD_) and treatment frequency of animal daily doses (TF_ADD_) from respectively the Danish cattle database (DCDB) and the VetStat Database

**Fig. 7:**
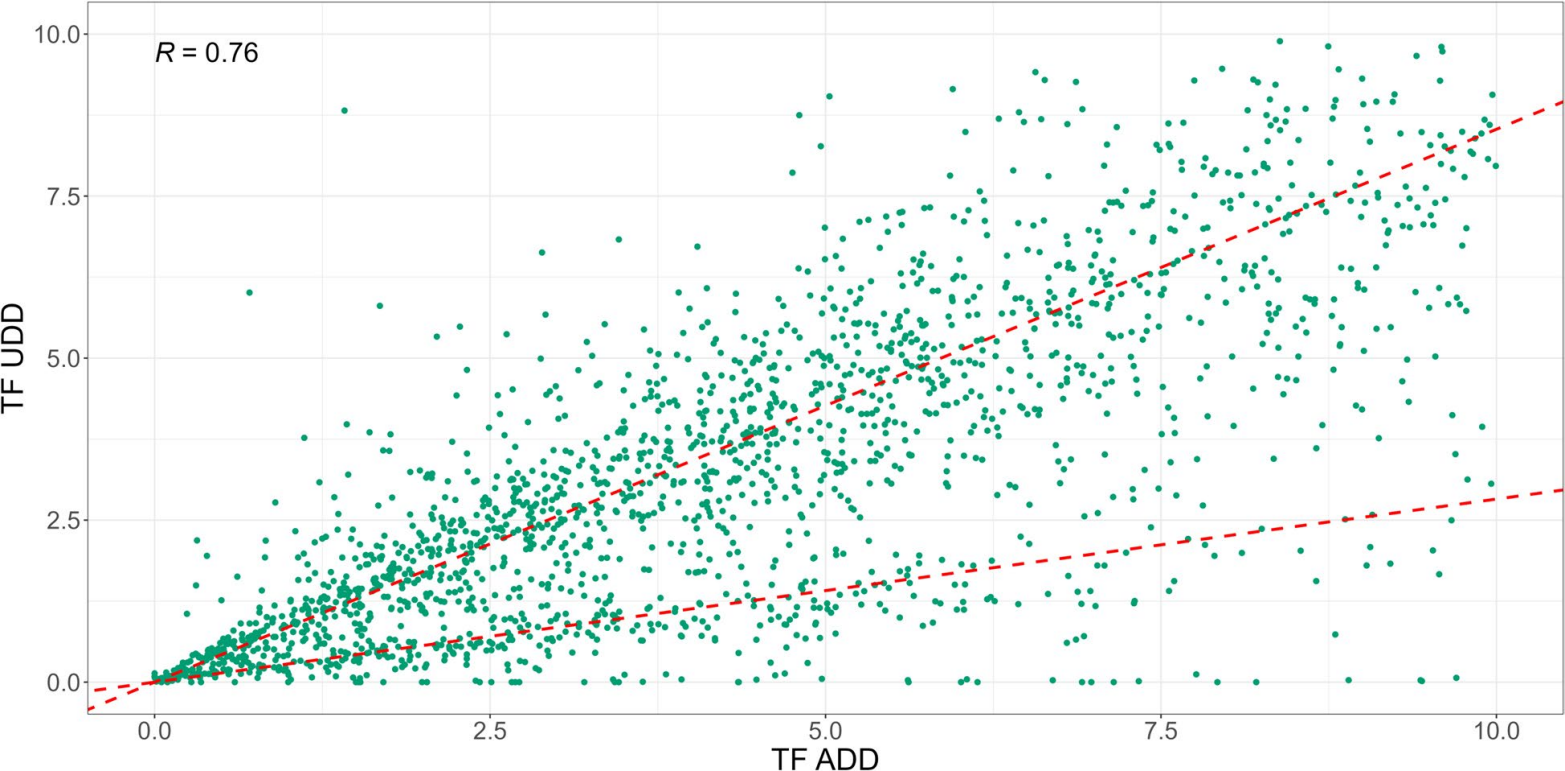
Spearman correlation plot of the farm level differences for the Udder prescription group in treatment frequency of used daily dose (TF_UDD_) and treatment frequency of animal daily doses (TF_ADD_) from respectively the Danish cattle database (DCDB) and the VetStat Database. Limits on both axes are set to (0,10), enabling visualization of systematic differences in the correlations for lower TF_UDD_ and TF_ADD_ observations. Dashed lines indicate correlation of each pattern, when dividing the data 3:1 (TF_ADD_:TF_UDD_). The R-value is the Spearman correlation coefficient for all the observations displayed, indicating a strong correlation between TF_UDD_ and TF_ADD_

The Spearman correlation analysis (Fig. 6) was conducted to evaluate the relationship between TF_UDD_ and TF_ADD_ per prescription group per farm. Each Spearman correlation coefficient identifies the correlation between TF_UDD_ and TF_ADD_ on farm level for each prescription group, showing a strong correlation for the Udder and Reproduction prescription groups. A weak, or no, correlation was found for the remaining prescription groups. The majority of the observations belong to the Udder prescription group, where two linear patterns are revealed for smaller TF_UDD_ and TF_ADD_ values. Two distinct linear patterns for the prescription group “Udder” when limiting TF_UDD_ and TF_ADD_ to a maximum of 10 each were observed (Fig. 7). One pattern exhibited a slope of 1 (TF_UDD_) to 3 (TF_ADD_), indicating a consistent threefold difference between TF_UDD_ and TF_ADD_. The other pattern showed a slope of 1:1, suggesting a more proportional relationship between TF_UDD_ and TF_ADD_.

## Discussion

The Bland–Altman plot based on the six different prescription groups in Fig. 3 shed light on the agreement between TF_UDD_ and TF_ADD_ in the context of animal antibiotic administration. The presence of five groups near the “difference = 0” line indicates a high level of concordance between TF_UDD_ and TF_ADD_ within those groups. This suggests that the prescription practices and dosing protocols employed in those groups lead to similar TF_UDD_ and TF_ADD_ values. However, the observation that all groups either occur with a difference around the 0-line or below, suggests a consistent bias towards lower TF_UDD_ values compared to TF_ADD_ values. When considering the 95% confidence interval, most of the groups fell within the limits, indicating acceptable agreement between TF_UDD_ and TF_ADD_. The notable exception is the Udder prescription group, which fell below the lower CI limit and exhibited the highest difference value. This group demonstrated a significant discrepancy between TF_UDD_ and TF_ADD_ (Table 1), indicating a potential issue or inconsistency in the prescription or administration of antibiotics. Further investigation is warranted to identify the factors contributing to this deviation and to assess its potential implications for treatment effectiveness or dosage optimization. Treatment of mastitis with both local and systemic drugs simultaneously (combination treatment) is commonly occurring among Danish veterinarians [21]. If a farmer or a veterinarian dose the medicine in accordance with the summary of product characteristics, a combination of local and systemic treatment may count as more than one ADD in VetStat, depending on the dosage and animal weight. Meanwhile, the same combination treatment will only be counted as one UDD in the DCDB since the diagnosis, day and animal treated is identical for the two products. This will result in a systematically lower TF_UDD_ for farms using this treatment approach and a Spearman correlation coefficient below one. When reporting count-based measures of AMU such as UDD used in this study, it should be addressed how combination treatments are reported. The ATCvet Bland–Altman plot (Fig. 4) shows the same pattern as for the prescription groups. The notable outlier is also related to udder treatments, this being the ATCvet code QJ01CE09, procaine benzylpenicillin. This antimicrobial product is commonly used in dairy cattle production for systemically treatment of gram-positive mastitis infections. It belongs to the classification QJ01 (antibacterial for systemic use) [21]. For TF_ADD_ and TF_UDD_ per farm, we observed that the Bland–Altman plot (Fig. 5) showed a consistent pattern with most of the 2,197 farm level comparisons between the TF_ADD_ and TF_UDD_ falling below the difference = 0 line. This indicates that the TF_UDD_ values tended to be lower than the corresponding TF_ADD_ values. This finding suggests a potential systematic difference between the two variables. The systematic discrepancy in prescriptions for Udder contributes significantly and further analyses should be conducted. The previously mentioned antimicrobial product procaine benzylpenicillin is not limited to use for mastitis infections. It is also frequently prescribed by veterinarians for use in joint infections according to VetStat data. If a farm uses the same product for udder and joint infections, it would be relevant to investigate whether the product is used on-farm for the correct prescription group. If this is not the case it would affect the analysis, which focuses on the prescription groups separately. If a product prescribed for udder is used for joint infections, our analyses can overestimate of the difference between TF_UDD_ and TF_ADD_.

In addition to use of medicine prescribed for a different prescription group, it might also be relevant to look at medicine prescribed for a different age group. It could for example be relevant to investigate whether medicine prescribed for cows and adult cattle is also routinely used for young stock or calves on-farm. To summarise, it is important to look at the equivalency between prescription pattern and use pattern when comparing count-based and dose-based measures of AMU.

We also observed instances where TF_UDD_ values exceeded TF_ADD_ values, suggesting deviations above zero. These variations might be attributed to specific dosing protocols or other factors influencing the measurements. For farms with systematically high number of UDDs compared to ADDs the breed of cattle might play a significant role. Currently, the majority of Danish dairy cows are large breeds, predominantly Danish Holstein, while Jersey breed cows account for 12% of the population (as retrieved from the DCDB). For products administered according to body weight, farms with Jersey cows might have systematically fewer calculated ADDs. This could be further analysed utilising data from the DCDB on breed of each individual animal treated. Generally, the differences in the fixed body weight, 600 kg, used in VetStat and the body weight estimated by the farmer or veterinarian when calculating dosage for a treatment should be addressed in further studies. It could be an important factor contributing to differences seen between the two measures of TF.

Outliers with a significant difference in values and a low average value were identified. This highlights the presence of exceptional cases that deviate substantially from the overall pattern. These extreme observations may be indicative of measurement errors, data entry issues, or unique circumstances requiring further investigation. Overall, our findings at the farm level revealed a consistent bias towards lower TF_UDD_ values compared to TF_ADD_ values (Table 2).

The presence of two distinct linear patterns in the Spearman plot (Figs. 6 and 7) indicates different correlations between TF_UDD_ and TF_ADD_ across farms. The pattern with a slope of 1:3 (Fig. 7) suggests a consistent threefold difference between TF_UDD_ and TF_ADD_. This discrepancy may be attributed to combination treatments and potential missing or grouped registrations in the Danish Cattle Database (DCDB). It is plausible that antibiotic treatments of individual animals carried out over multiple days for the same diagnosis are only registered on the first day of treatment in DCDB. Such grouped registrations could contribute to the observed 1:3 relationship between UDD and ADD, indicating an underestimation of the actual UDD values in the DCDB. On the other hand, the pattern with a slope of almost 1:1 suggests a more balanced and proportional relationship between UDD and ADD. Farms exhibiting this pattern may have better registration practices or different treatment and dosage protocols that lead to a more accurate representation of UDD in the DCDB. This should be further investigated while also addressing the issue of combination treatments of mastitis. In addition to the issues mentioned above, waste of antibiotics at farms and missing recording of UDD on farm level should also be addressed in further analyses.

The findings in our study raise important considerations regarding the structure, reliability, and completeness of data in the DCDB. Further investigations are necessary to understand the underlying factors contributing to these distinct patterns highlighted in the present study and to address potential data limitations, ensuring the accuracy and completeness of the recorded information in the DCDB. Future studies on AMU should account for the source and structure of the data, as these may impact the results significantly. Also, when databases holding AMU are constructed, it is important to be aware of the pitfalls that might occur due to differences in prescription practices and management practices.

This study is based exclusively on registry data, and records cannot be validated beyond what is feasible through thorough data cleaning.

## Conclusion

Our comparison of UDD and ADD provides valuable insights into antimicrobial usage at the farm level. We demonstrate high correlations between UDD and ADD, with a notable exception for udder treatments, where some farms appear with only 1/3 UDD compared to ADD suggesting an underreporting due to registration, reporting, or treatment practices.

## Data Availability

We do not have the authority to share the data used for analysis.

## Abbreviations

AB: Antibiotics
ADD: Animal daily dose
AMR: Antimicrobial resistance
AMU: Antimicrobial usage
ATCvet: Anatomical therapeutic chemical classification system for veterinary medicinal products
CI: Confidence interval
DCDB: Danish cattle database
DDDvet: Defined daily doses for animals
DVFA: Danish veterinary and food administration
M2: Modul 2 extension
PCU: Population correction unit
SPC: Summary of product characteristics
TF: Treatment frequency
TF_ADD_: Treatment frequency of animal daily doses
TF_AMU_: Treatment frequency for antimicrobial usage
TF_UDD_: Treatment frequency of used daily doses
UDD: Used daily doses
VASC: Veterinary advisory service contract
VetStat: Danish VetStat database
WHO: World Health Organization
